# The Domain of Medical Police

**Published:** 1862-07

**Authors:** 


THE DOMAIN OF MEDICAL POLICE. (Abstract of a Paper read before the New York Sanitary Association, Feb. 6, 1862.) By LOUIS ELSBERG, M.A., M.D.

This is a well written pamphlet of 16 pages, in which the writer sets forth the objects and advantages of a judicious Medical Police, for ferreting out and removing the causes of disease. We have room only, for the following paragraph in relation to *Quarantine*, viz.:—

"In the protection of localities, it is believed that the most desirable quarantine is a *dirt quarantine.* 'Annihilate or exclude *dirt*, *i.e.*, putrefiable animal and vegetable detritus, and no *migratory* or *malignant* morbific poison can sustain its existence even if its cause be imported to the spot!' Thus it is maintained that, by sanitary reform, ALL MALIGNANT EPIDEMICS ARE PREVENTABLE; and it is interesting to know that this has been proved, even as regards plague, once the most destructive of all pestilences, and looked upon as the very *type* of *contagion*! It has now, so to speak, died out in those cities of the East to which it was formely a *frequent visitant*, if not an endemic. Long ago it was finally excluded from all the parts of *Western Europe*, *not* by quarantine, but by improved *local* SANITARY ARRANGEMENTS! Its fountain-head, the great Oriental capital, *Cairo*, before the time of Mehemed Ali, many times lost tens of thousands of inhabitants by its visitations. From here it spread, devastating over Egypt and the world! The viceroy named, and his son, Ibrahim Pacha, (though unaware of the stupendously beneficial results of the undertaking,) removed the hills between Boulaq and the mouth of the Nile, and transformed an immense swamp in the heart of the city, and the *receptacle* of all its filth, into a park, (or olive and fruit-bearing pleasure garden—the 'Esbekiah.') Other local sanitary arrangements were enforced, and now CARIO (*and with it, all humanity*) IS FREE FROM PLAGUE!

